# Supplementary material for: Evidence for secondary thrombotic microangiopathy in COVID-19
Source: medRxiv. 2020 Oct 23:2020.10.20.20215608. Preprint. [Version 1] doi: 10.1101/2020.10.20.20215608 (PMC7587832; doi:10.1101/2020.10.20.20215608)
Supplement: 1 [file NIHPP2020.10.20.20215608-supplement-1.pdf]

medRxiv preprint doi: https://doi.org/10.1101/2020.10.20.20215608; this version posted October 23, 2020. The copyright holder for this preprint (which was not certified by peer review) is the author/funder, who has granted medRxiv a license to display the preprint in perpetuity.
It is made available under a CC-BY 4.0 International license .

Supplementary Figures and Tables:

Supplementary Figure 1) Correlations amongst various endothelial activation and coagulation parameters

Supplementary Figure 2) Correlation of various hemolysis, endothelial activation, and coagulation parameters to Schistocyte Count.

Supplementary Figure 3) Correlations amongst various lab values measured to assess hemolysis and/or coagulopathies.

Supplemental Table 1) Thrombotic Events and Anticoagulation Treatment of 181 Patients with COVID19 Stratified by ADAMTS13 Activity Level

Supplementary Figure 4) Markers of coagulation, endothelial activation, or hemolysis stratified by the occurrence of a thrombotic event

Supplementary Figure 5) Lactate dehydrogenase and VWF antigen stratified by anticoagulation use.

Supplementary Figure 6) Correlation of D-Dimer with other classic markers of Disseminated intravascular coagulation (DIC) within 72 hours of admission.

medRxiv preprint doi: https://doi.org/10.1101/2020.10.20.20215608; this version posted October 23, 2020. The copyright holder for this preprint
(which was not certified by peer review) is the author/funder, who has granted medRxiv a license to display the preprint in perpetuity.
It is made available under a CC-BY 4.0 International license .

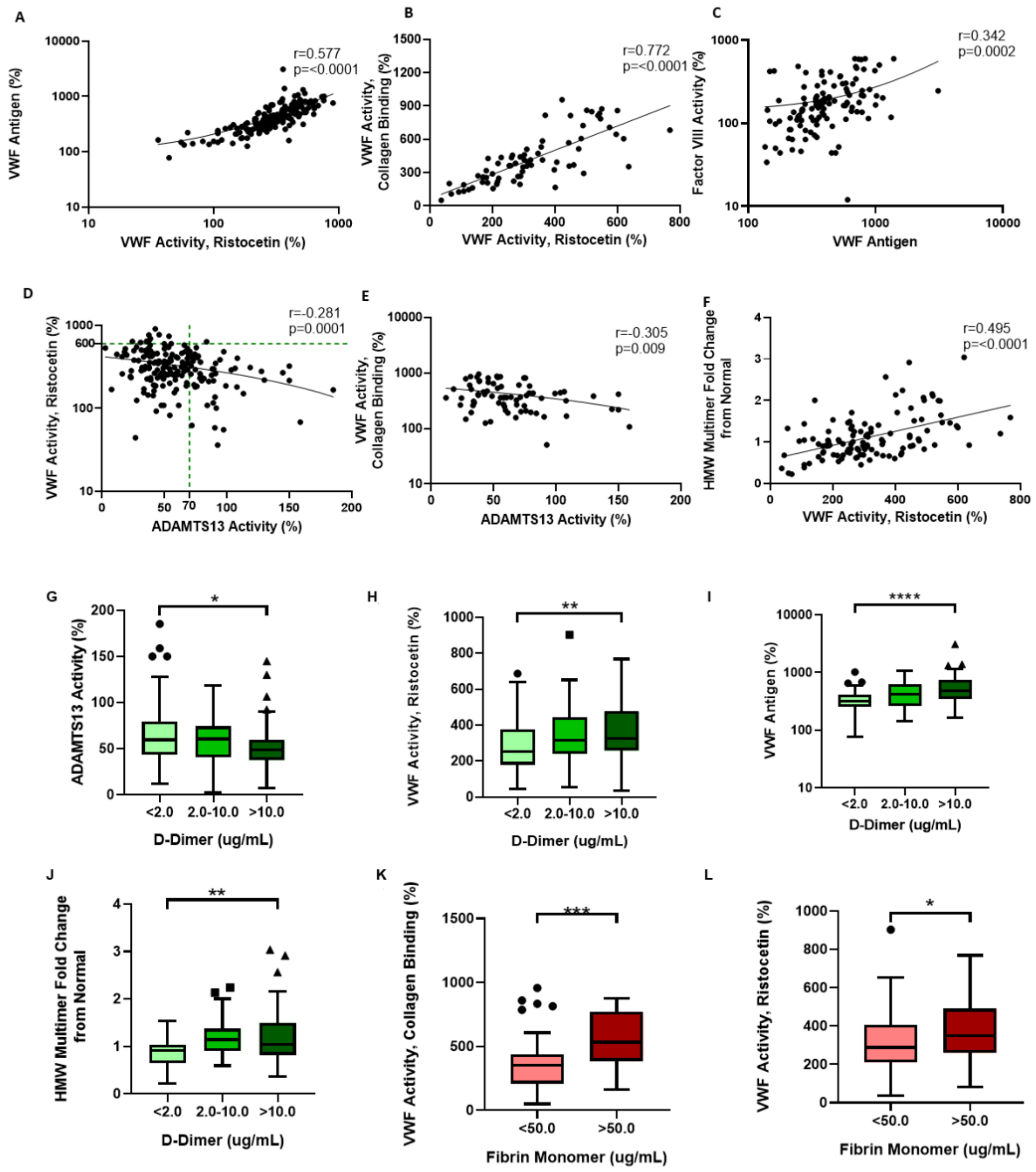

Supplementary Figure 1) Correlations amongst various endothelial activation and coagulation parameters. The Pearson's coefficient (r), p-value, and trendline is shown for graphs A-F. All 181 patients are represented unless otherwise stated. A) Scatter plot showing positive correlation between VWF Antigen and VWF Ristocetin activity. B) Scatter plot showing positive correlation between VWF Collagen Binding activity and VWF Ristocetin activity. This only includes patients for whom an ELISA for VWF Collagen Binding activity was completed (n=72) C) Scatter plot showing slight positive correlation between Factor VIII activity and VWF Antigen (n=116) D) Scatter plot showing negative correlation between VWF Ristocetin activity and ADAMTS13 Activity. Almost all (10/11) VWF activity levels greater than 600% occur in patients with ADAMTS13 levels less than 70%. E) Scatter plot showing negative correlation between VWF Collagen Binding activity and ADAMTS13 Activity (n=72). F). Scatter plot showing positive correlation between VWF Ristocetin activity and the fold change of each patient's HMW multimer size compared to the HMW multimer size of the normal pooled plasma control. This only includes patients for whom multimer western blots were ran (n=115). G-L. Within each box plot, the horizontal line indicates the median, the outside bars indicate the 25th and 75th percentile, individual dots indicate outlier points, and asterisk represent the p-value from a one-way ANOVA (if three values), or two tailed t-test (if two values). The asterisk indicates significance as follows: *$p < 0.05$, **$p < 0.01$, ***$p < 0.001$, and **** $P<0.0001$. The Box Plot shows G) ADAMTS13 Activity (n=181) H) VWF Ristocetin activity (n=181) I) VWF Antigen (n=181) J) fold change in High Molecular Weight Multimer compared to normal (n=115), stratified by low (<2ug/mL), medium (2.0-10.0 ug/mL), or high (>10ug/mL) D-dimer concentration. Generally, each lab parameter became more abnormal in the medium and high D-Dimer stratification compared to the low stratification. The Remaining box plots show K) VWF Collagen Binding Activity (n=72) and L) VWF Ristocetin activity (n=181), stratified by low (<50ug/mL) or high (>50ug/mL) Fibrin Monomer concentration. Generally, each lab parameter was more abnormal in the high Fibrin Monomer stratification compared to the low stratification.

medRxiv preprint doi: https://doi.org/10.1101/2020.10.20.20215608; this version posted October 23, 2020. The copyright holder for this preprint (which was not certified by peer review) is the author/funder, who has granted medRxiv a license to display the preprint in perpetuity.
It is made available under a CC-BY 4.0 International license .

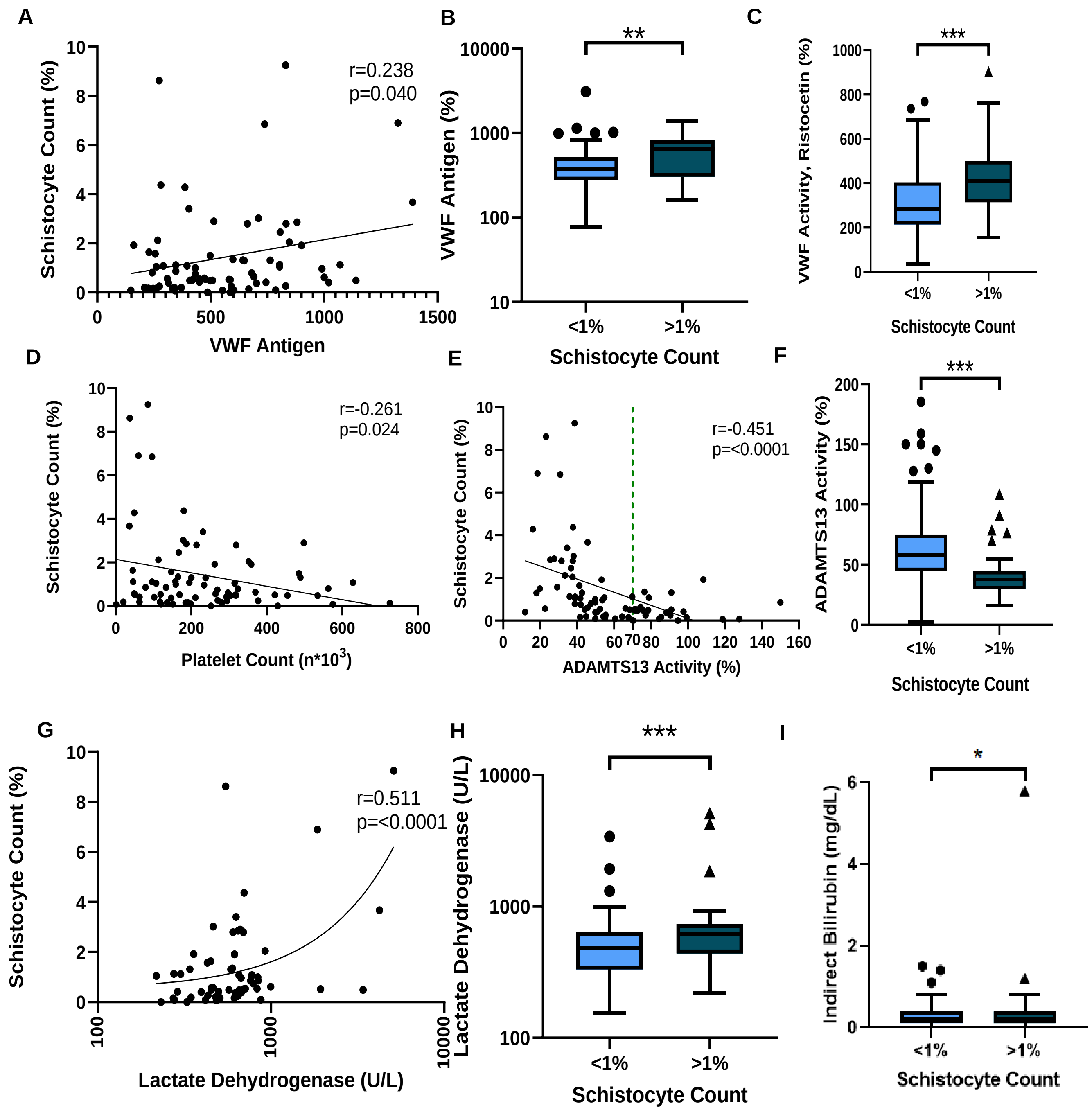

Supplementary Figure 2) Correlation of various hemolysis, endothelial activation, and coagulation parameters to Schistocyte Count. For each scattter plot (A, D, E G), the Pearson's coefficient (r), p-value, and trendline is shown. Only patients for whom a CBC was flagged as abnormal within three days of when the sample was taken and therefore could be specifically quantified are included in the scatter plots (n=73). A) Scatter plot showing positive correlation between the Schistocyte count and VWF antigen. D) Scatter plot showing negative correlation between the Schistocyte count and Platelet count. All cases of schistocyte counts greater than 4% occurred in patients with a platelet count less than 200,000/ml E) Scatter plot showing negative correlation between the Schistocyte count and ADAMTS13 activity. All cases of schistocyte counts greater than 2% occurred in patients who had ADADMTS13 activity less than normal 70% (dotted green line). G) Scatter plot showing positive correlation between the Schistocyte count and lactate dehydrogenase. For each box plot (B, C, F, H, I), the horizontal line indicates the median, the outside bars indicate the 25th and 75th percentile, individual dots indicate outlier points, and the asterisk represent the p-value from a two tailed t-test. The asterisk indicates significance as follows: *$p < 0.05$, **$p < 0.01$, ***$p < 0.001$, and **** $P<0.0001$. All patients are included in the box plots (n=181) unless otherwise noted, with patients without an abnormal CBC flag within three days of the sample assumed to have <1% schistocyte count (see methods). The box plots show B) VWF antigen C) VWF Ristocetin activity F) ADAMTS13 H) Lactate Dehydrogenase (n=158) and I) Indirect Bilirubin levels (n=171) stratified by low (<1%) or high (>1%) schistocyte count.

medRxiv preprint doi: https://doi.org/10.1101/2020.10.20.20215608; this version posted October 23, 2020. The copyright holder for this preprint (which was not certified by peer review) is the author/funder, who has granted medRxiv a license to display the preprint in perpetuity.
It is made available under a CC-BY 4.0 International license .

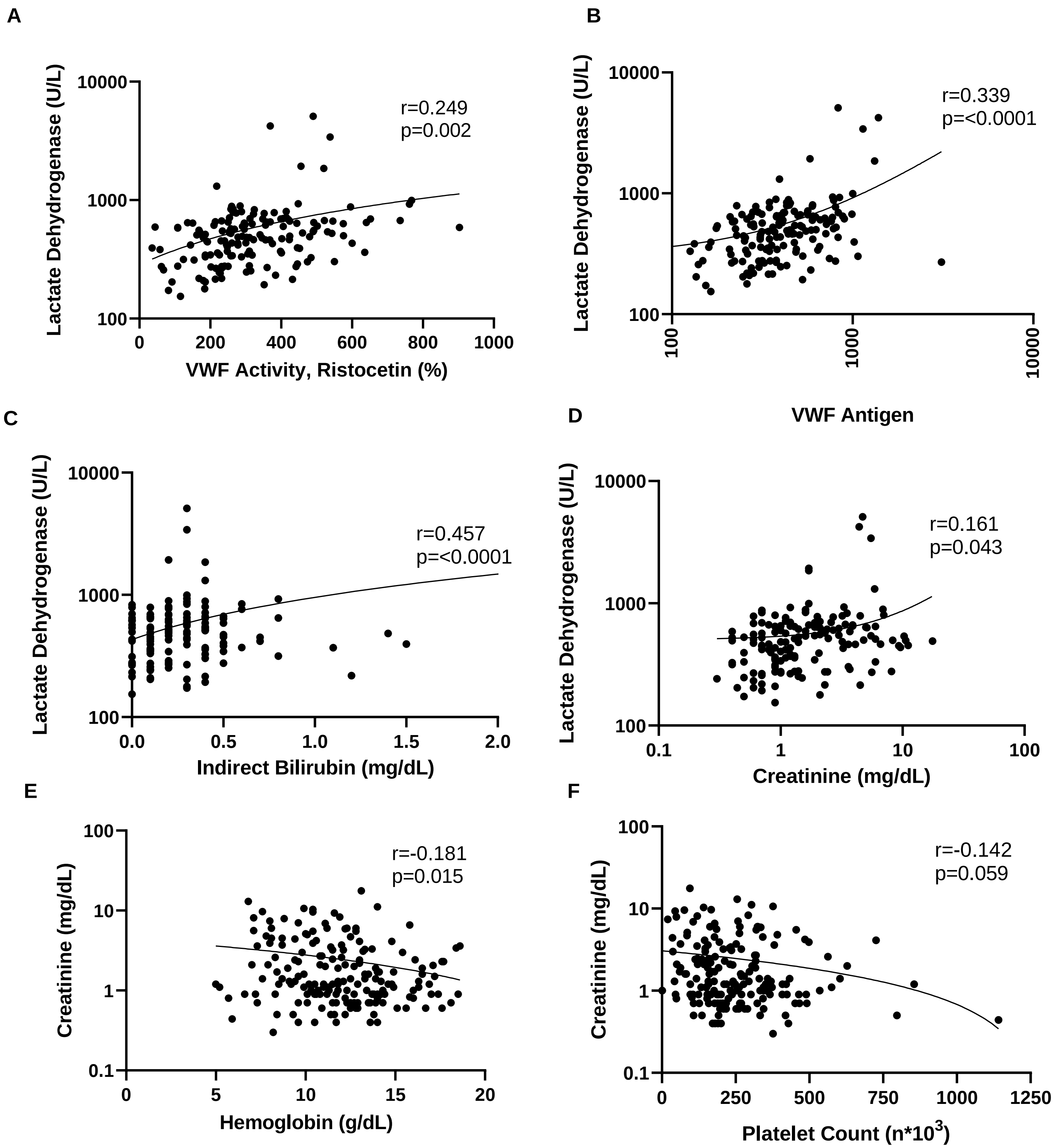

Supplementary Figure 3) Correlations amongst various lab values measured to assess hemolysis and/or coagulopathies. The Pearson's coefficient (r), p-value, and trendline is shown for each graph. All 181 patients are represented in each plot unless otherwise stated. A) Scatter plot showing positive correlation between lactate dehydrogenase level and VWF Ristocetin activity (n=158) B) Scatter plot showing positive correlation between lactate dehydrogenase level and VWF antigen (n=158). C) Scatter plot showing positive correlation between lactate dehydrogenase level and indirect bilirubin. Only cases for which an LDH and bilirubin measurements were taken within 48 hours of the sample are included (n=158) D) Scatter plot showing positive correlation between lactate dehydrogenase level and creatinine level (n=158) E) Scatter plot showing negative correlation between creatinine level and hemoglobin level F) Scatter plot showing negative correlation between creatinine level and platelet count.

medRxiv preprint doi: https://doi.org/10.1101/2020.10.20.20215608; this version posted October 23, 2020. The copyright holder for this preprint (which was not certified by peer review) is the author/funder, who has granted medRxiv a license to display the preprint in perpetuity.
It is made available under a CC-BY 4.0 International license .

Supplementary Table 1) Thrombotic Events and Anticoagulation Treatment of 181 Patients with COVID19 Stratified by ADAMTS13 Activity Level

Characteristics, median [IQR] or n (%)	Low ADAMTS13 Activity (<70%) (n=129[a])	Normal ADAMTS13 Activity (>70%) (n=52[a])	p
**ADAMTS13 Activity (%) [70-110]**	46.1 [36.7, 55.4]	88.71 [76.3, 100.2]	<0.001
**Schistocyte Count (%) [<0.5]**	1.06 [0.52, 2.54]	0.48 [0.16, 0.59]	<0.001
**Age**	68.0 [59.0, 78.0]	64.0 [49.8, 73.0]	0.02
**Sex (M)**	77 (59.7)	29 (56)	0.63
**Mortality**	71 (55.0)	19 (37)	0.02
**Continuous Renal Replacement Therapies**	19 (14.7)	6 (12)	0.75
**Hemodialysis Use**	22 (17.1)	7 (14)	0.71
**Thrombotic or Clotting Event within entire Hospital Admission, n (%)**			
**Thrombosis**	23 (17.8)	13 (25)	0.37
• **Deep Venous Thrombosis**	14 (10.9)	10 (19)	0.21
• **Pulmonary Embolism**	3 (2.3)	1 (2)	1.000
• **Arterial Thrombosis**	3 (2.3)	2 (4)	0.63
• **Stroke**	2 (1.6)	0 (0)	1.000
**Ex Vivo Clotting**	10 (7.8)	1 (2)	0.18
**Thrombotic or Clotting Event within 7 Days of ADAMTS13 Activity Measurement, n (%)**			
**Thrombosis**	16 (12.4)	4 (8)	0.44
• **Deep Venous Thrombosis**	10 (7.8)	3 (6)	0.76
• **Pulmonary Embolism**	1 (0.8)	0 (0)	1.000
• **Arterial Thrombosis**	3 (2.3)	1 (2)	1.000
• **Stroke**	2 (1.6)	0 (0)	1.000
**Ex Vivo Clotting**	7 (5.4)	0 (0)	0.20
**Anticoagulation[b], n (%)**			
**None**	80 (62.1)	34 (65)	0.67
**Prophylactic**	26 (20.2)	12 (23)	0.81
• **Heparin**	9 (7.0)	1 (2)	0.29
• **Enoxaparin**	10 (7.8)	4 (8)	1.000
• **Apixaban**	7 (5.4)	7 (14)	0.13
**Therapeutic**	23 (17.8)	6 (12)	0.41
• **Heparin**	1 (0.8)	1 (2)	0.49
• **Enoxaparin**	4 (3.1)	0 (0)	0.58
• **Apixaban**	9 (7.0)	3 (6)	1.000
• **Bivalirudin**	8 (6.2)	2 (4)	0.73
• **Warfarin**	1 (0.8)	0 (0)	1.000
**Anticoagulation before Thrombosis or Ex Vivo Clot[c], n (%)**			
**None**	13/30 (43)	5/13 (38.5)	1.000
**Prophylactic**	4/30 (13)	4/13 (31)	0.22
**Therapeutic**	11/30 (37)	4/13 (31)	1.000
**Change of Anticoagulation after [illegible]**	19/30 (63)	11/13 (85)	0.30

a. Unless otherwise stated
b. all patients within 48 hours before clot or ADAMTS13 measurement
c. Out of patients who experienced thrombosis or an ex vivo clot; 48 hours before clot

Abbreviations: IQR, interquartile range; ADAMTS13, a disintegrin and metalloproteinase with a thrombospondin type 1 motif, member 13

medRxiv preprint doi: https://doi.org/10.1101/2020.10.20.20215608; this version posted October 23, 2020. The copyright holder for this preprint (which was not certified by peer review) is the author/funder, who has granted medRxiv a license to display the preprint in perpetuity.
It is made available under a CC-BY 4.0 International license .

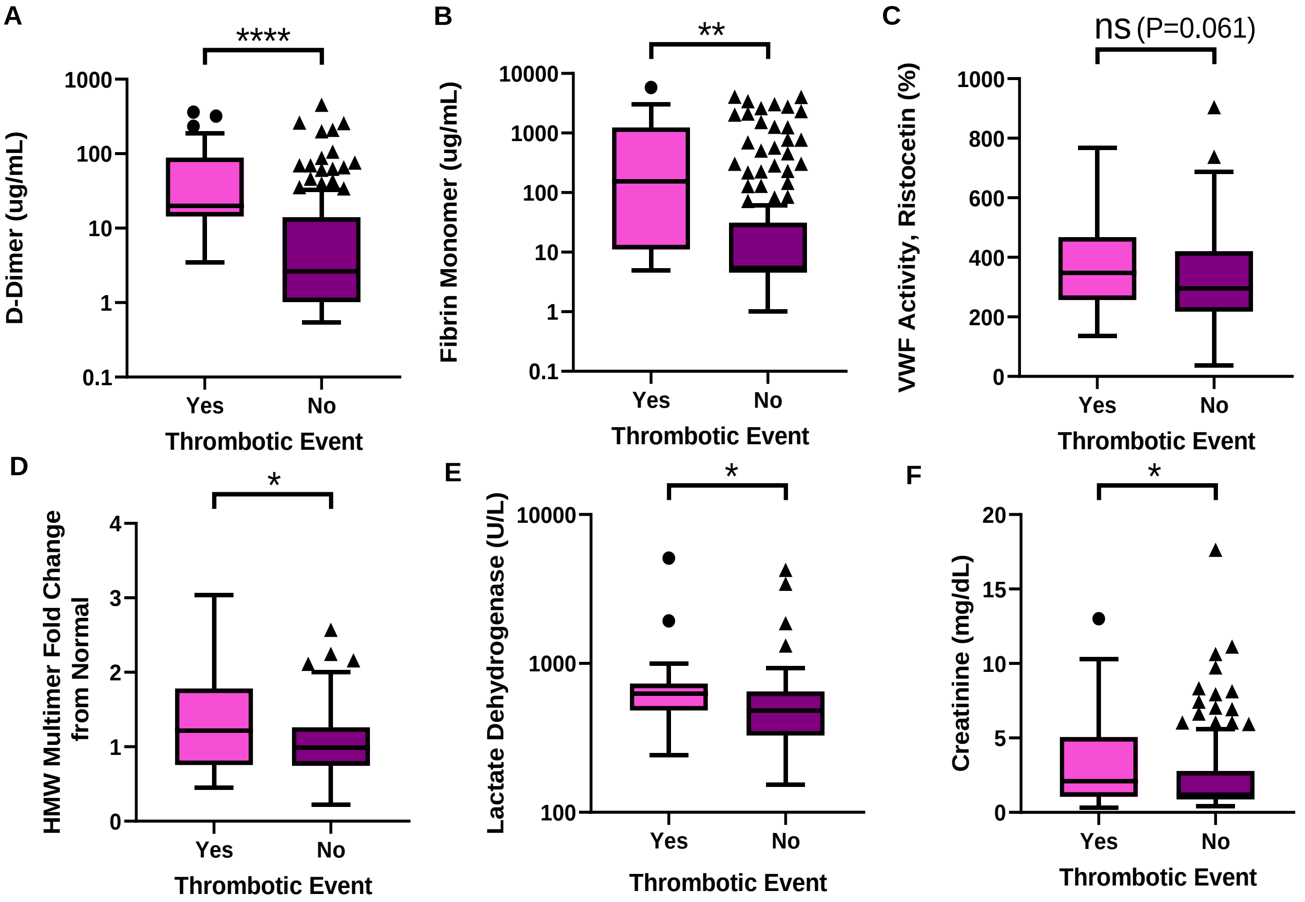

Supplementary Figure 4) Markers of coagulation, endothelial activation, or hemolysis stratified by the occurrence of a thrombotic event. We considered a thrombotic event to be either an occurrence of in vivo thrombosis if it was documented with radiographic imagining, or an ex vivo clot if it was reported in the patient's chart. All events within 7 days of the blood sample we used to measure the markers of coagulation and hemolysis were considered. Within each box plot, the horizontal line indicates the median, the outside bars indicate the 25th and 75th percentile, individual dots indicate outlier points, and asterisk represent the p-value from a two tailed t-test. The asterisk indicates significance as follows: *p < 0.05, **p < 0.01, ***p < 0.001, and **** P<0.0001. The Box Plots show A) D-Dimer B) Fibrin monomer C) VWF Ristocetin activity D) fold change of HMW multimer size compared to that of normal pooled plasma E) Lactate dehydrogenase or F) Creatinine level stratified by a thrombosis or clotting event within 7 days of the sample.

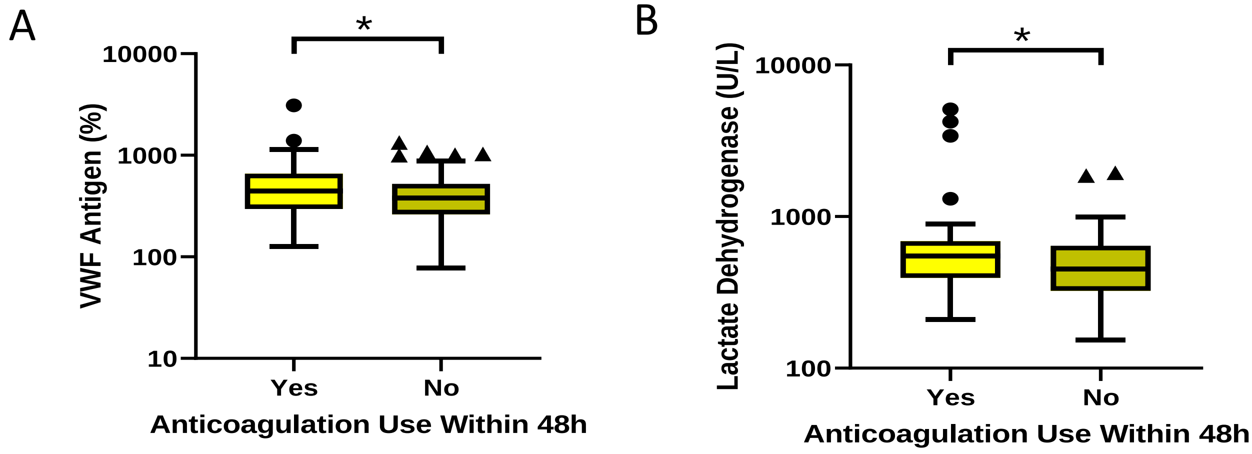

Supplementary Figure 5) VWF antigen and Lactate dehydrogenase stratified by anticoagulation use. Within each box plot, the horizontal line indicates the median, the outside bars indicate the 25th and 75th percentile, individual dots indicate outlier points, and asterisk represent the p-value from a two tailed t-test. The asterisk indicates significance as follows: *p < 0.05, **p < 0.01, ***p < 0.001, and **** P<0.0001. The Box Plots show A) VWF antigen and B) Lactate dehydrogenase stratified by anticoagulation use. A patient was considered to be on anticoagulation medication if it was administered at least 48 hours prior to when the sample was taken.

medRxiv preprint doi: https://doi.org/10.1101/2020.10.20.20215608; this version posted October 23, 2020. The copyright holder for this preprint
(which was not certified by peer review) is the author/funder, who has granted medRxiv a license to display the preprint in perpetuity.
It is made available under a CC-BY 4.0 International license .

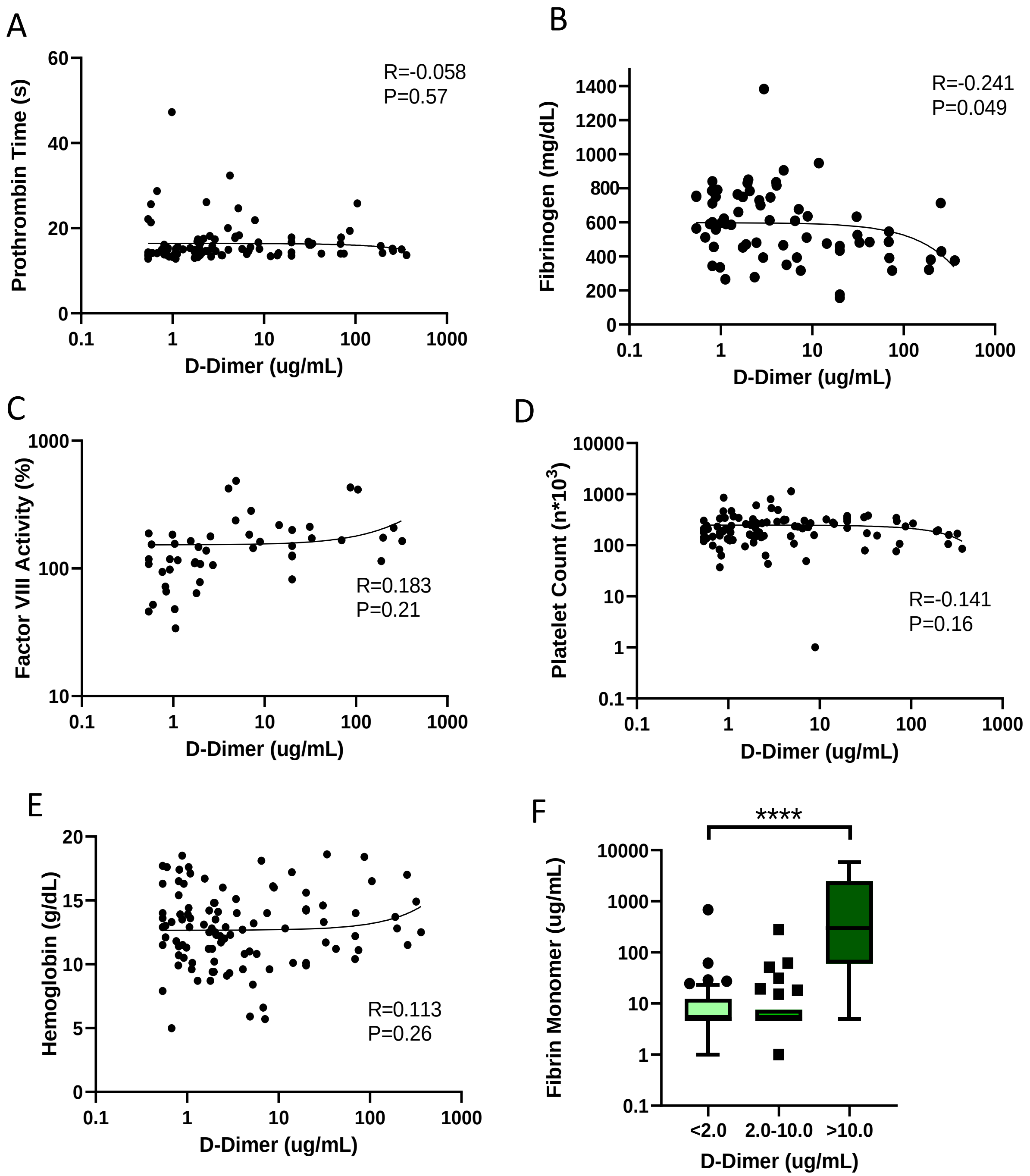

Supplementary Figure 6) Correlation of D-Dimer with other classic markers of Disseminated intravascular coagulation (DIC) within 72 hours of admission. The Pearson's coefficient (r), p-value, and trendline is shown for each graph. All 102 patients for whom an ADAMTS13 measurement was taken within 72 hours of admission are represented unless otherwise stated. A) Scatter plot showing no significant correlation between prothrombin time and D-Dimer B) Scatter plot showing slight negative correlation between fibrinogen and D-Dimer (n=67) C) Scatter plot showing no significant correlation between factor VIII activity and D-Dimer (n=48) D) Scatter plot showing no significant correlation between platelet count and D-Dimer E) Scatter plot showing no significant correlation between hemoglobin and D-Dimer F) Box plot of fibrin monomer stratified by low (<2ug/mL), medium (2.0-10.0 ug/mL), or high (>10ug/mL) D-dimer concentration. The horizontal line indicates the median, the outside bars indicate the 25th and 75th percentile, individual dots indicate outlier points, and asterisk represent the p-value from a one-way ANOVA. Four asterisks indicates the p value is <0.0001.
